# Memory for spatio-temporal contextual details during the retrieval of naturalistic episodes

**DOI:** 10.1038/s41598-021-93960-9

**Published:** 2021-07-16

**Authors:** Samy-Adrien Foudil, Claire Pleche, Emiliano Macaluso

**Affiliations:** 1 ImpAct Team, Lyon Neuroscience Research Center, Lyon, France; 2 Lyon Neuroscience Research Center (ImpAct Team), 16 avenue Doyen Lépinel, 69500 Bron, France

**Keywords:** Learning and memory, Cognitive neuroscience

## Abstract

Episodic memory entails the storage of events together with their spatio-temporal context and retrieval comprises the subjective experience of a link between the person who remembers and the episode itself. We used an encoding procedure with mobile-phones to generate experimentally-controlled episodes in the real world: object-images were sent to the participants' phone, with encoding durations up to 3 weeks. In other groups of participants, the same objects were encoded during the exploration of a virtual town (45 min) or using a standard laboratory paradigm, with pairs of object/place-images presented in a sequence of unrelated trials (15 min). At retrieval, we tested subjective memory for the objects (remember/familiar) and memory for the context (place and time). We found that accurate and confident context-memory increased the likelihood of “remember” responses, in all encoding contexts. We also tested the participants' ability to judge the temporal-order of the encoded episodes. Using a model of temporal similarity, we demonstrate scale-invariant properties of order-retrieval, but also highlight the contribution of non-chronological factors. We conclude that the mechanisms governing episodic memory retrieval can operate across a wide range of spatio-temporal contexts and that the multi-dimensional nature of the episodic traces contributes to the subjective experience of retrieval.

## Introduction

The traditional approach to studying behavior consists in isolating specific processes by using simple and highly stereotyped stimuli, deprived of any context. This may include presenting participants with simple visual shapes and measuring perceptual or motor performance or using lists of isolated words to study specific memory processes^1,2^. While this approach provided us with an impressive amount of knowledge about cognitive functions, and their underlying neural bases, impoverished laboratory conditions may miss many relevant factors that contribute to behavior in the real world. Researchers have sought to tackle this by using more naturalistic stimuli, including static images of real-world scenes, dynamic videos and virtual reality settings^3–5^. Static images comprise objects-in-context and allow studying how contextual information and prior knowledge about the spatial layout of real-world environments affect the allocation of perceptual/attentional resources^6,7^ or memory processes^3,8^. Dynamic stimuli, such as movies, add the temporal dimension and can entail a coherent flow of information over time that, again, has been found to constrain perceptual and memory processes^9,10^. Virtual reality permits to further enrich the experimental context and to include active interactions between the participant and the context^4,11^. Here we take this a step further by introducing an innovative approach based on mobile-phone technology to investigate memory for experimental events occurring during the everyday life of the participants (i.e. images of objects received on the mobile-phone).

The study of episodic memory is one of the fields where moving from simple, stereotyped paradigms to complex and naturalistic conditions is particularly relevant. A defining characteristic of episodic memory is that it entails the storage of events together with their spatio-temporal context^12,13^. Episodic retrieval includes not only memory for “what” happened, but also for the associated contextual details, e.g. “where” and “when” the event happened. These associations can be studied using simple stimuli (e.g. spatial-location and/or list-position of words presented on a computer screen), but in natural conditions these dimensions are tightly coupled and contribute to shaping the coherent spatio-temporal continuum that characterizes everyday life. A second key feature of episodic memory concerns the intimate relationship between the person who experiences the event and the episode itself^13–16^. Using simple stimuli, previous studies sought to target this aspect by manipulating the ownership status of the encoded item^17,18^ or by asking the participants to perform simple actions during encoding ("enactment")^19–21^. These manipulations have been found to enhance the subjective relevance of the encoded episode and to strengthen memory performance. Nonetheless, they only weakly approximate the personal experience of acting in the real world.

Virtual reality (VR) provides us with means of investigating episodic memory in conditions that entail active behavior within a coherent spatio-temporal continuum and can result to high-levels of personal engagement. Episodic memory studies in VR typically involve a first phase when the participants encode a series of items/events in the environment, followed by a memory retrieval phase testing for some aspects of the memorized events. Overall, encoding in VR has been associated with an increase of memory performance^22–24^; see also^25^ for review.

Beside overall performance, VR protocols allowed addressing the more specific issue of how the characteristics of the encoding experience affect the quality of the subsequent retrieval. The latter includes, on the one hand, the availability of information about the encoding context (e.g. the “where and when” of a given episode) and, on the other hand, the subjective experience of recollection. In the framework of dual-process models of episodic memory^26,27^, it has been suggested that the binding of the multiple elements of an episode may contribute to recollection, which would entail the access to the specific spatio-temporal context associated with the memorized episode^28–30^. For example, Jebara et al.^31^ investigated how “active vs. passive” VR navigation affected subsequent event- and context-retrieval and their memory status. The results in young adults showed better event memory and what-where-when binding in a passive-*planning* condition compared with active-*driving* condition. These somewhat surprising results indicate that active behavior in complex environments (here, active-*driving*) may actually reduce memory encoding. In this study, the memory status of the episode (*Remember* vs. *Know*) was unaffected by the encoding condition (but, note that the different encoding conditions did affect performance in an older group of participants). Persson et al.^32^ investigated the relationship between the memory status of the events (*Remember* vs. *Familiar*) and the ability to retrieve contextual information. Memory encoding entailed a small VR environment (2 rooms, with windows facing on an external courtyard) and the results showed that context details (i.e. the outdoor weather) could be reliably retrieved only when the memory status of the object was *Remember.* By contrast, memory for the temporal position of the different encoding events was available also when the episode was *Familiar*. Using a far larger environment (48 rooms), Horner and colleagues^33^ also reported some uncoupling between temporal-order performance and context-memory. Specifically, temporal-order memory was above chance and was modulated by the presence of physical boundaries (i.e., a change of room), while spatial memory (where/in-what-room a given object was seen) was instead at chance level. Accordingly, previous studies using VR demonstrated that several aspects of the encoding context, such as the spatio-temporal characteristics of the environment and the cognitive demands of the task, can affect memory performance and modulate the relationship between the subjective memory status of the what-event (recollection vs. familiarity) and memory for the associated where/when-sources.

However, encoding in VR still entails substantial differences compared to any real-world situation. In particular, the real physical position of the participant is fixed in space (i.e. the participant sits in the laboratory throughout encoding) leading to a crossmodal miss-match between the visual input and body position. This may weaken the participants' subjective experience of the spatial context and reduce the binding of the episodic elements^25^. Furthermore, VR protocols forcibly entail relatively short encoding periods (i.e., max a few hours). This is likely to constrain how the temporal context is represented and, thus, how the timing of specific events is embedded within such representation^34^.

**Figure 1:**
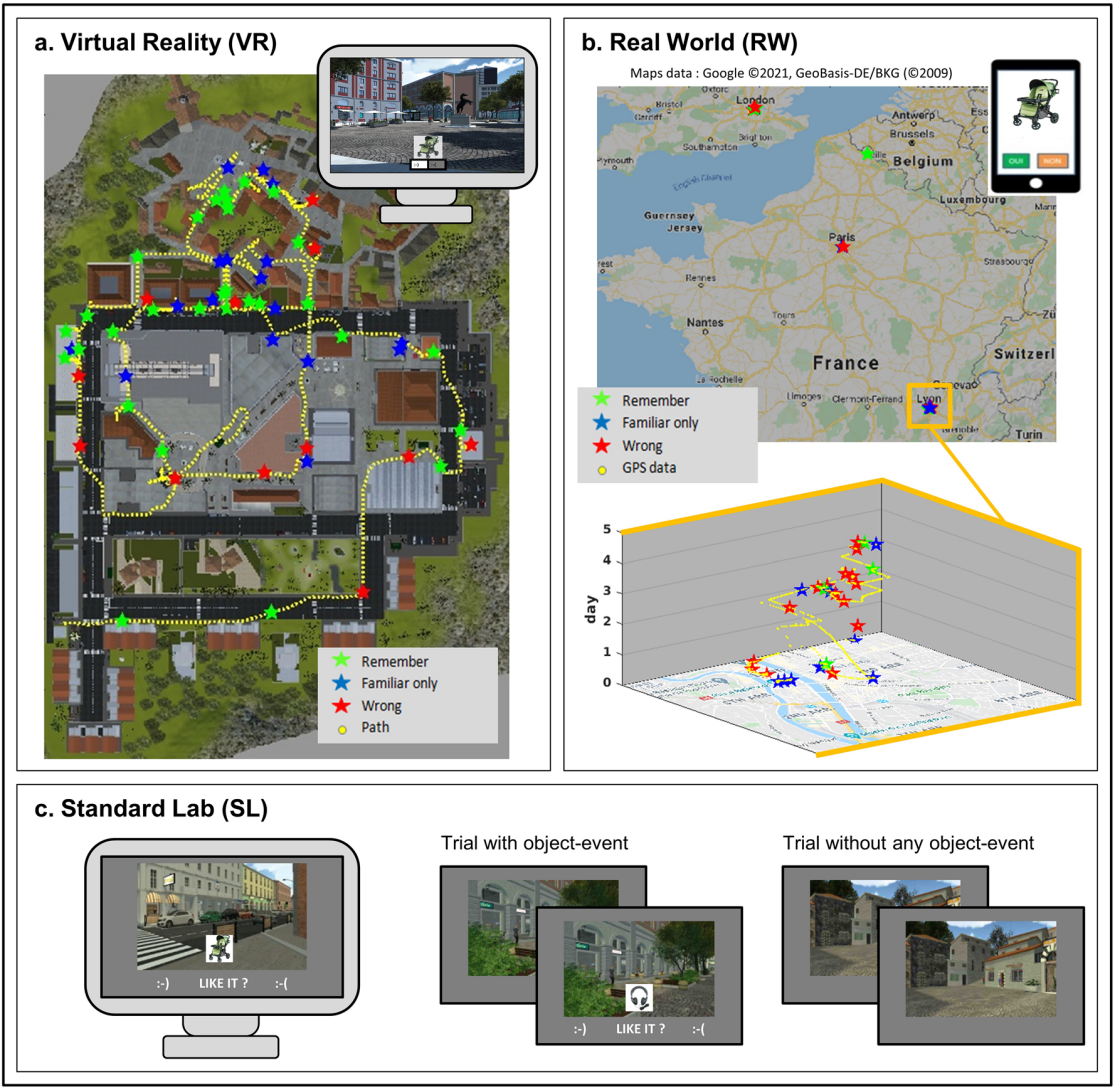
Encoding protocols. (**a**) The virtual reality protocol (VR) entailed active exploration of a virtual town for 45 min. The main task of the participants was to collect specific items in virtual shops. At unpredictable intervals, pictures of objects appeared in the bottom part of the screen and the participants performed a “like/dislike” judgment (see inset, top-left). The main panel shows the path of one participant (yellow dotted-line) and the locations where the memory probes were presented (stars, with the different colors coding whether the object was subsequently retrieved as *Remember*, *Familiar* or wrongly categorized as a *New* object). (**b**) The real-world protocol (RW) entailed the encoding of the memory probes via a dedicated mobile-phone system, over a period of up to several weeks (see also Table 1). The participants received the object-images on the mobile-phone and responded with a “like/dislike” judgment. The position of the participant was geolocalised via the GPS functionality of the mobile-phone. The panel presents the data of one participant, who traveled in different cities and countries during the encoding phase. The bottom part of the panel shows a close-up view of the participant's path in the area of Lyon during a 5 days period (see z-axis). (**c**) Illustration of the Standard Laboratory protocol (SL) that comprised a sequence of unrelated trials presented on the computer screen. Half of the trials included arbitrarily paired object- and place-images ("Trial with object-event"). The other half of the trials included only the place-images ("Trial without any object-event). When the trial included the object-image, the participant performed the “like/dislike” judgment. The place-images showed views of the virtual town (cf. **a**).

**Figure 2:**
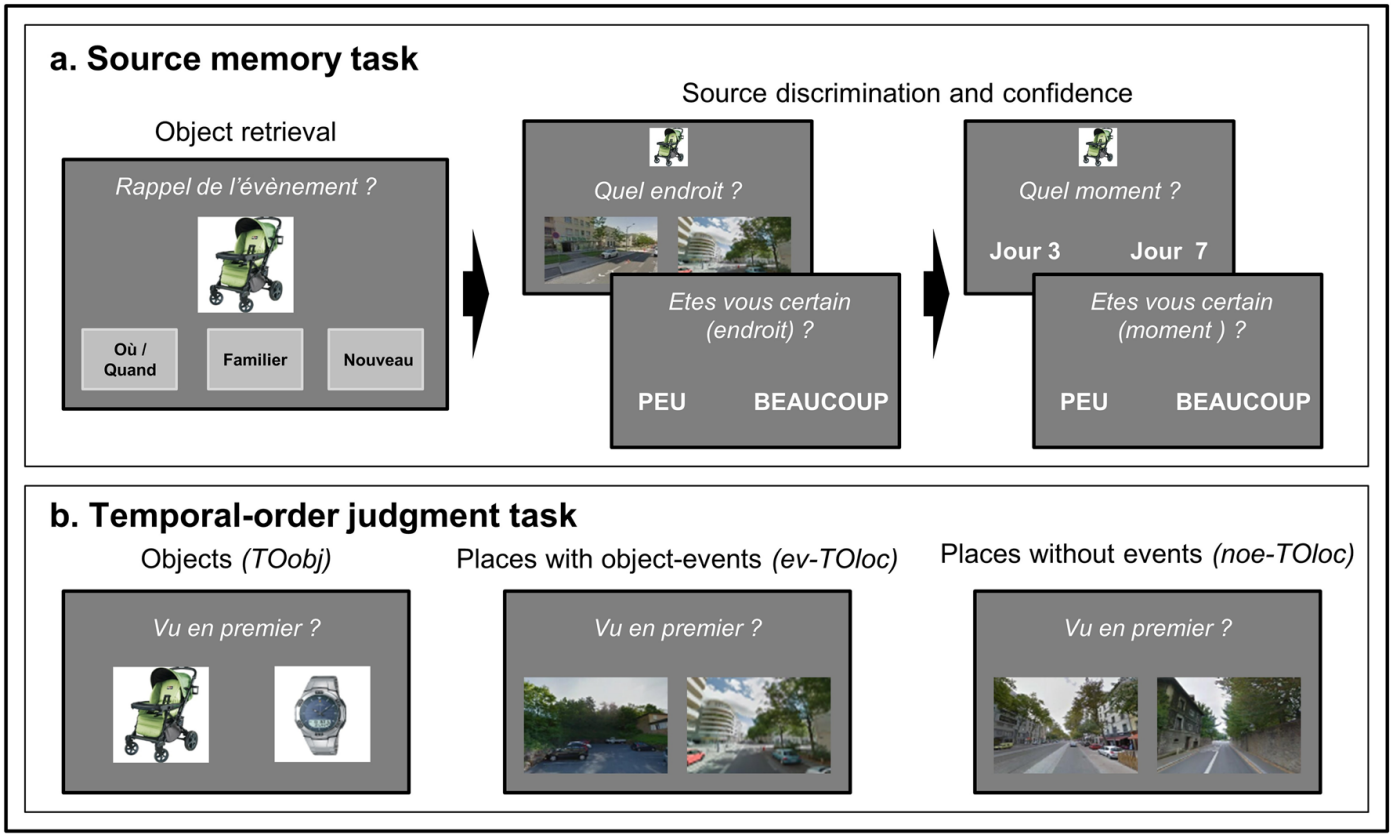
Retrieval tasks. (**a**) Illustration of the phases of the explicit source retrieval task. The task began with the presentation of the object-probe and the participant reported whether they had seen the object during the encoding phase and whether they could remember the place/time of that event. We label the three possible responses as *Remember*, *Familiar* or *New*. If the object was seen at encoding and correctly recognized as old (i.e. *Rem* or *Fam* response)*,* the trial continued with a sequence of questions concerning the time/place-context. The place-test comprised a 2-alternative forced-choice discrimination task involving the presentation of an image associated with the place where the object was presented during encoding, plus a foil place-image. For the VR and SL protocols the place-images corresponded to the participant's view at encoding, while for the RW protocol the images were obtained from Google-images based on GPS coordinates and phone orientation. A confidence question followed the place-image discrimination ("how sure are you? very much/little"). The time-test also entailed a 2-alternative forced-choice, now comprising two time windows. One of the two windows included the moment when the object was presented. A confidence question followed the time-discrimination. The presentation order of the place and time source-tests was randomized across trials. (**b**) Example trials of the temporal-order judgment task. All trials included the presentation of two images side-by-side and the task of the participant was to report what image referred to the event that happened earlier during the encoding. In two separate blocks, the images comprised either the memorized object (*TOobj*), or images of places (*TOloc*). The place-images were either associated with an object-event during encoding (*ev-TOloc*) or referred to seen/visited places but where no object-event took place (*noe-TOloc*). The *ev-TOloc* and *noe-TOloc* trials were randomized within the *TOloc* block. The task's instructions were displayed in French.

To address these limitations, we propose an innovative strategy to generate experimentally-controlled episodes in the everyday life of the participants (see also “Discussion” section, for related studies using portable cameras^35–37^). Using mobile-phone technology, we tracked the geolocation of the participants in the real world for relatively long periods, up to several weeks (RW protocol, see Fig. 1b; Table 1). Based on specific spatio-temporal constraints (see Suppl. Material), we sent images of objects to the participants' phone and we recorded the real-world place and time of each of these events. We then tested the memory status of the items (what-object, *Remember*/*Familiar*/*New*), as well as the accuracy and confidence for the associated context (i.e. where-place and when-time), see Fig. 2a. Moreover, we employed a temporal-order judgment task to investigate the temporal representation of events encoded in such a large scale spatio-temporal context (Fig. 2b). The same retrieval tasks were also administered to two separate groups of participants, who encoded the same set of object-images either in a highly interactive large-scale virtual environment (VR protocol, Fig. 1a) or using a “standard” encoding procedure that included a series of unrelated trials each comprising the pairing of an object and a place-image on a computer screen (SL: standard laboratory, Fig. 1c).

**Table 1:** Main spatio-temporal characteristics of the three encoding contexts.

	Virtual reality (VR)			Real world (RW)			Standard Laboratory (SL)		
	Mean	Min	Max	Mean	Min	Max	Mean	MIN	Max
Encoding	00:00:42:25	00:00:42:24	00:00:42:38	10:04:17:38	02:19:28:12	17:01:57:46	00:00:14:51	00:00:14:14	00:00:15:08
Retention	01:00:43:08	00:22:02:06	01:05:51:50	03:21:20:29	00:02:13:04	12:16:08:30	00:00:18:34	00:00:11:31	00:00:23:08
Path length	4.5	2.8	5.3	972.8	99.4	2495.0	*n.a*	*n.a*	*n.a*

*Encoding*: time between the encoding of the first and the last object. *Retention*: time between the encoding of the last object and the retrieval of the first object (explicit source retrieval task). Times are in the format “DAYS:HOURS:MINUTES:SECONDS”. *Path length*: The participants' total movement during the encoding phase. For the VR protocol the values correspond to “virtual” kilometers in the virtual town. For the RW protocol, the path length corresponds to kilometers in the real world. The SL protocol did not involve any real or virtual movement of the participant. *VR* virtual reality, *RW* real world, *SL* standard laboratory, *n.a.* not applicable.

The three protocols vastly differed in terms of the spatial–temporal scale of the encoding context. The participants travelled over distances of up to hundreds of kilometers during encoding in the real world (RW), while their position was fixed in lab for VR and SL. On the temporal side, both encoding durations and retention intervals lasted up to several weeks in RW vs. minutes-to-hours in VR and SL (see Table 1). Because of this, our analyses sought primarily to evaluate whether specific retrieval mechanisms held across protocols, rather than seeking to interpret any difference in overall memory performance. Specifically, we carried out logistic regressions analyses to predict the memory status of the what-objects (*Remember*/*Familiar)* from the accuracy and the confidence for the corresponding where/when-context; and we used a model of temporal similarity^38^ to test for scale invariance in temporal-order retrieval. In particular, the temporal similarity scores account both for the encoding times and for the time intervals between encoding and retrieval (see “Methods” section), allowing us to compare the three protocols with different ranges of intervals.

## Results

### Explicit source retrieval

The main aim of the source retrieval task was to test how the availability of place/time source information contributes to the subjective memory status of the encoded objects (*Rem* vs *Fam*), in the three different encoding contexts. Each trial started with the presentation of the picture of an object. The task of the participant was to choose one of 3 possible responses: (a) I have seen this object and I have some memory of the place/time when this happened; (b) The object is familiar but cannot remember when/where I saw it; (c) I have not seen the object. We label these three response-types as “Remembered” (*Rem*), “Familiar” (*Fam*) and “New” (*New*). If the object was seen during encoding and the participant responded *Rem* or *Fam*, the trial went on testing for when/where source-memory and confidence (see Fig. 2a).

**Figure 3:**
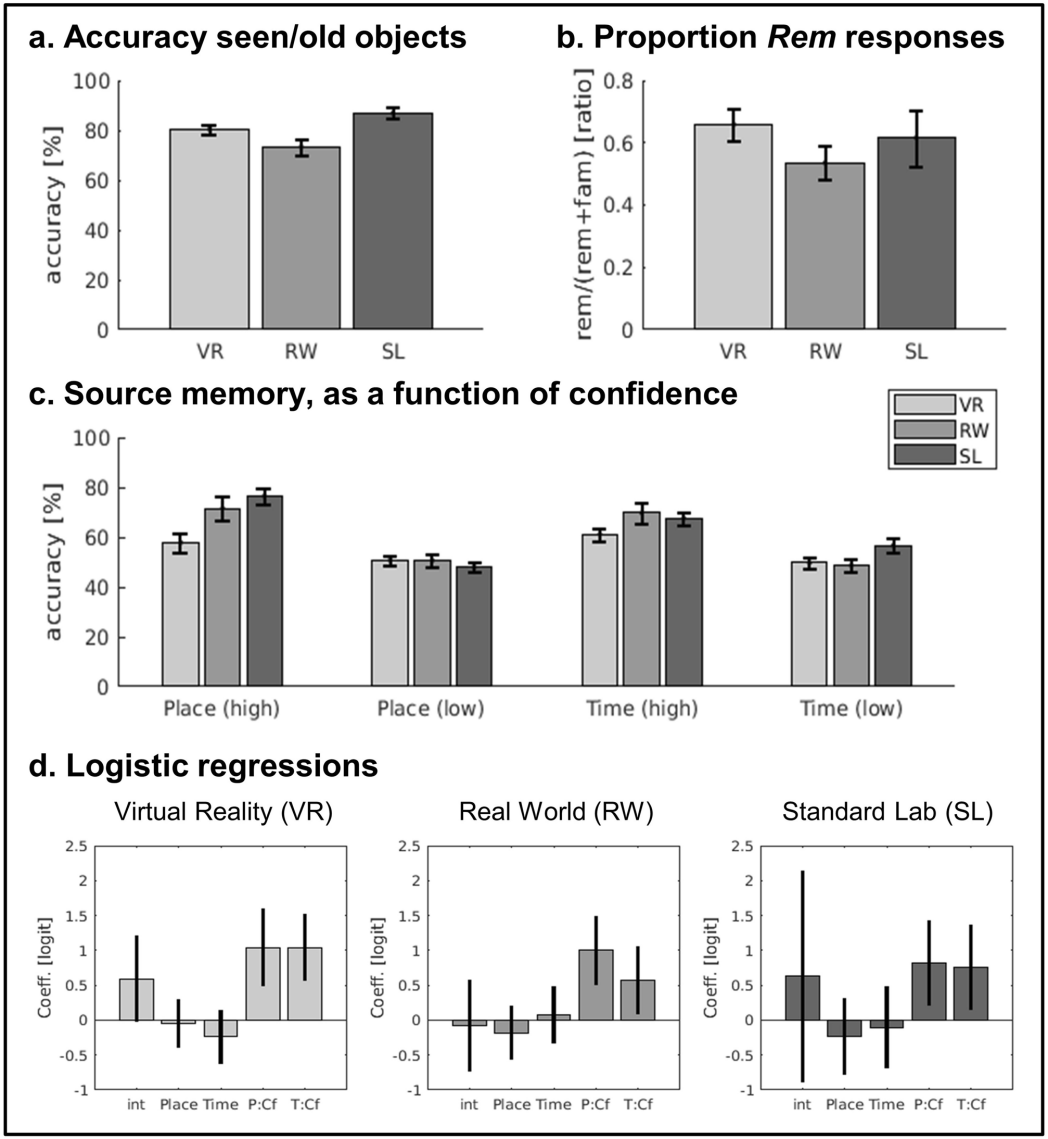
Results of the explicit source task. (**a**) Mean accuracy (± SEM) of the recognition of old/seen objects, irrespective of the subjective memory status (*Remember* or *Familiar*). (**b**) Proportion of *Remember* responses (± SEM) for correctly recognized old/seen objects. (**c**) Mean accuracy (± SEM) of the place- and time-source discrimination, as a function of confidence (high/low). (**d**) Regression coefficients (logit, ± 95% confidence intervals) of the binomial regressions seeking to predict changes of the proportion of *Remember* responses to the what-object (dependent variable) using the accuracy and confidence of the subsequent source-memory judgments. The regressions included 5 predictors: the intercept (*int*), the response to the place- and time-discrimination, irrespective of confidence (*Place* and *Time*; coding 1/0, correct/wrong) and the effect of correct and confident source judgments (*P:CF* and *T:CF*; coding 1 correct source-discrimination followed by high confidence rating and 0 for all the other response combinations). The results showed that correct and confident source-discrimination of places and times lead to a significant increase of the probability of *Remember* responses, in all three encoding contexts. VR/RW/SL: virtual reality, real world and standard laboratory encoding protocols.

First, we report to the overall object- and source-memory performance. A between-groups ANOVA tested the effect of the encoding context on the participants’ memory for old/seen objects, irrespective of *Rem/Fam* responses (see Fig. 3a). This revealed a significant effect of experiment (F(2,57) = 7.23; *p* = 0.002; see also Table S1). Subsequent post-hoc tests (Tukey HSD) revealed that the accuracy in RW was significantly lower than in SL (*p* = 0.001). We then tested whether the encoding context affected the subjective memory status of the correctly recognized seen objects. The ratio of *Rem*/(*Rem* + *Fam*) correct responses were submitted to a between-groups ANOVA. The ANOVA did not reveal any significant difference between the three experiments (F(2,57) = 0.84; *p* = 0.438; see Fig. 3b).

Next, we tested the effect of the encoding context on memory for the when/where sources, as a function of the confidence of the source discrimination. The discrimination accuracy data were submitted to a mixed-ANOVA including the 3 factors: “source” (place/time: within-subject), “confidence” (high/low: within-subject) and “experiment” (VR/RW/SL: between-groups). The ANOVA revealed a main effect of confidence (F(1,56) = 68.02; *p* < 0.001), a main effect of experiment (F(2,56) = 5.66; *p* = 0.006), a significant interaction between confidence and experiment (F(2,56) = 3.29; *p* = 0.044), as well as a significant 3-way interaction (F(2,56) = 5.20; *p* = 0.008), see Fig. 3c. The participants could reliably retrieve the place and time sources, when they reported to be confident about their response: the accuracies for all the high-confident conditions were significantly above chance-level (all *p* < 0.05; see also Table S1). By contrast, the only low-confidence condition leading to performance above chance was the time-test in SL (cf. last bar in the plot of Fig. 3c), contributing to the observed 3-way interaction.

To address our main question about the contribution of source memory to the subjective memory status of the objects, mixed-effects logistic regressions sought to predict the probability of *Rem* responses (vs. *Fam*, considering only correctly recognized old/seen trials) based on the accuracy and the confidence of the source discrimination. The regression models included 4 predictors coding for place/time accuracy (irrespective of confidence) and for correct-source discrimination with high-confidence (vs. all the other combinations of accuracy and confidence). The results revealed a significant contribution of source-memory to the object memory status, but only when the sources were discriminated correctly and with high-confidence. Both confident-place and confident-time memory led to an increase of the probability to respond *Rem* (vs *Fam*) and this held for all three encoding contexts (see Fig. 3d). For confident-place the significance levels were: < 0.001 (VR), < 0.001 (RW), 0.010 (SL); and for confident-time they were: < 0.001 (VR), 0.022 (RW), 0.016 (SL). None of the predictors coding for accuracy, irrespective of confidence, were significant (all *p*'s > 0.2) An additional set of logistic regressions tested whether trials including both confident-place and confident-time judgments lead to any further increase of the probability of *Rem* responses, but this was not significant in any of the three contexts (all *p* values > 0.05).

### Temporal-order judgments

The main aim of the second retrieval task was to investigate the representation of temporal distances in our 3 protocols, which entailed vastly different temporal scales (see Table 1). In two separate blocks, the participants judged the temporal-order of two events, based either on pairs of object-images (*TOobj* task) or pairs of place-images (*TOloc* task). Further, in *TOloc,* the two images showed either places where an object-event had taken place during encoding (*ev-TOloc*); or two places that were also visited/seen, but without any object-event (*noe-TOloc*), see Fig. 2b. Across trials, the temporal distance at encoding between the two to-be-judged events was varied parametrically. Temporal distances were transformed into temporal similarity scores (TS) allowing us to test for invariance in temporal-order retrieval across the three encoding protocols.

**Figure 4:**
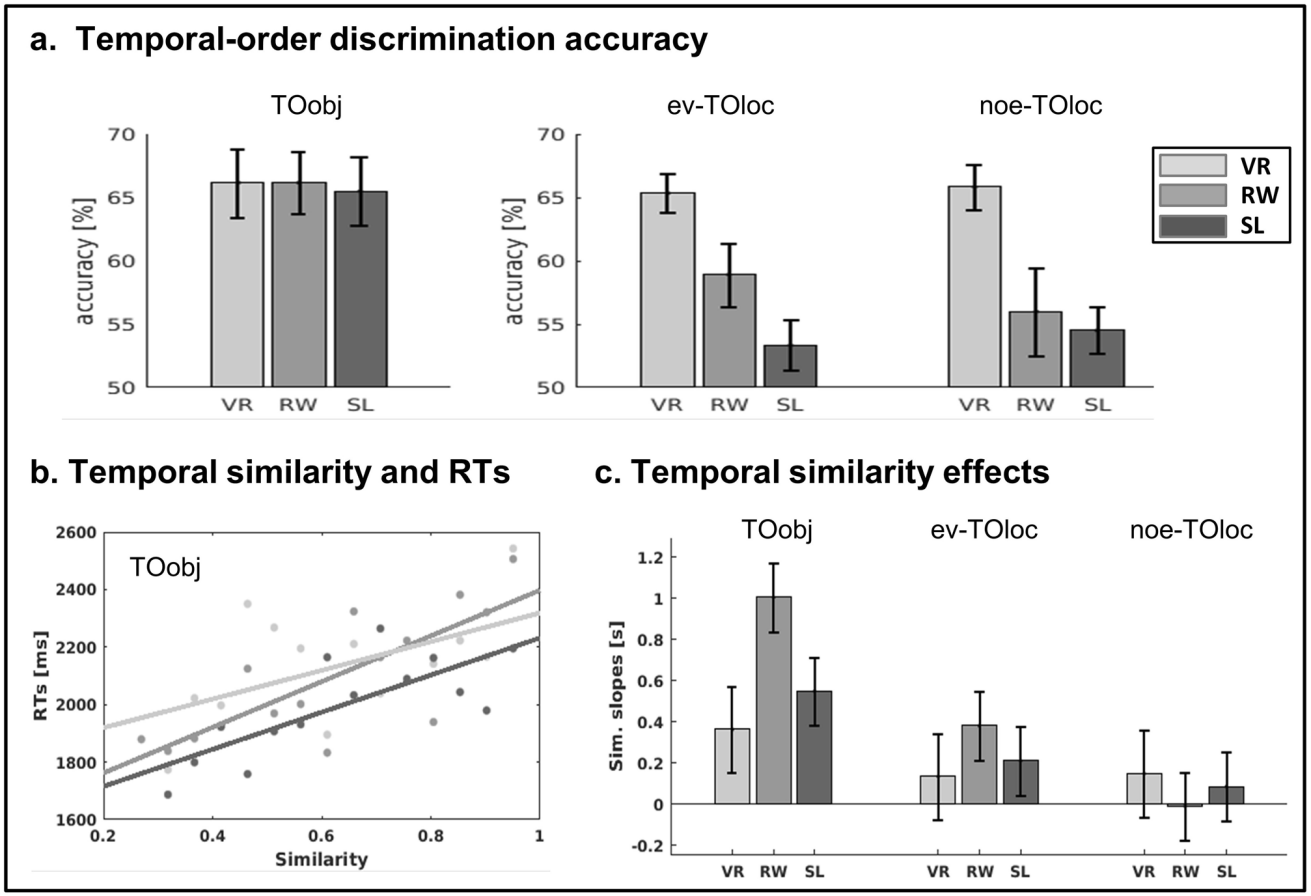
Results of temporal-order judgment task. (**a**) Mean accuracy (%, ± SEM) of the temporal-order discrimination task using object-images as retrieval cues (*TOobj*). (**b**) Mean accuracy (%, ± SEM) of the temporal-order discrimination task using place-images as retrieval cues (TOloc). The first 3 bars show the accuracy for places where an object-event took place (*ev-TOloc*), while the 3 bars on the right show the accuracy for trials comprising place-images without any object-event (*noe-TOloc*). (**c**) For illustration purposes only, the *TOobj* trial-specific data were averaged in 15 bins, allowing us to display the regression lines for the three protocols (VR, RW, SL). The regression lines show that the RTs increased with increasing temporal similarity, in all 3 experiments. (**d**) Average slopes (± SEM) of the regressions between temporal similarity and RTs, now calculated at the level of the individual participant and displayed separately for the different types of retrieval cues (*TOobj, ev-TOloc, noe-TOloc*) and the three encoding protocols (VR, RW, SL). The data analysis revealed a general effect of temporal similarity across experiments, supporting scale invariance of order retrieval, but also a significant effect of cue-type indicating that factors other than chronological distance also contributed to temporal order retrieval, see “Discussion” section.

First, we report the overall accuracy of the temporal-order judgments. A between-groups ANOVA tested whether the encoding context (VR, RW, SL) modulated the overall accuracy of the temporal-order retrieval using the object-cues. This was not significant (F(2,57) = 0.02, *p* > 0.9; see Fig. 4a, left panel), suggesting that information about the presentation order of the objects was available irrespective of context. Next, we asked whether the participants could retrieve the order of the visited/seen places and whether the fact of having received—and responded to—an object-event in these places affected the temporal-order performance. A mixed-ANOVA with the factors “experiment” (VR, RW, SL) and place “with/without” events (*ev-TOloc, noe-TOloc*) revealed only a main effect of experiment (F(2,57) = 10.13; *p* < 0.001). Post hoc tests (Tukey HSD) revealed that VR accuracy was higher than both RW (*p* = 0.009) and SL (*p* < 0.001), while RW and SL did not differ significantly from each other (*p* > 0.3), see Fig. 4a, panel on the right.

Our main analyses examined the influence of temporal similarity on the retrieval reaction times (RTs). We used the SIMPLE model^38^ to compute the similarity between the two events to be judged on each trial and correlated this with the corresponding RT on a trial-by-trial basis for each subject, condition (*TOobj*, *ev-TOloc*, *noe-TOloc*) and experiment (VR, RW, SL). Figure 4b illustrates the influence of temporal similarity on RTs for the *TOobj* condition. For statistical inference, the regression slopes were estimated at the level of the individual participant using trail-specific similarity values and RTs. The resulting slopes (beta values) were submitted to a mixed-ANOVA with the factors “experiment” and “cue-condition”, see Fig. 4c. This highlighted a significant main effect of “condition” (F(2,114) = 7.00; *p* = 0.001), with steeper slopes in *TOobj* compared with the two *TOloc* conditions. The ANOVA did not reveal any significant main effect or interaction related to the factor “experiment” (*p*'s > 0.3), suggesting that analogous order-retrieval processes operated across temporal scales ranging from seconds (SL) to weeks (RW). We sought to confirm this scale invariance by submitting the average similarity slopes first to a one-sample T-test including all three experiments (t(59) = 5.84, *p* < 0.001), and then to a Bayesian analysis to evaluate the null-hypothesis of no difference between the experiments (see also^5^). The results showed “extreme evidence” in favor of the null-model (Bayes factor = 0.009, with JZS prior), supporting scale invariance.

## Discussion

We investigated episodic memory retrieval following encoding in three contexts that comprised vastly different spatio-temporal scales and levels of naturalism (see Fig. 1; Table 1). In the framework of the what-where-when dimensions of episodic memory, we tested participants' memory for the encoded what-item (pictures of objects) and for the corresponding where-place and when-time context (Fig. 2a), as well as for the participants' ability to retrieve temporal order information based on what-object or where-place cues (Fig. 2b). The results revealed a set of general principles linking the different episodic dimensions, irrespective of encoding context. These included the proportion of objects retrieved with a *Remember* memory status (vs. *Familiar*, Fig. 3b), the contribution of source memory and confidence to this (cf. logistic regressions, Fig. 3d), the role of the temporal similarity between two episodes when retrieving their temporal order, as well as the impact of the cueing dimension on the latter (what-object vs. where-places, see Fig. 4c). By using an innovative real-world encoding protocol, these findings contribute to the understanding of binding processes in episodic memory and support proposals of temporal scale invariance in long-term memory.

A first objective of the current study was to assess the impact of the encoding context on item-memory, source-memory, and their relationship. In the framework of dual-process models of episodic memory it has been suggested that the availability of source information should contribute to recollection as opposed to familiarity^39^. Studies employing arbitrary associations between the item and the sources (e.g. using the item's color, the stimulus’ position on the screen or in a list, as the context/source) provided us with mixed results (e.g. see^40^, reporting chance-level source performance for familiar items, vs. high proportion of correct source judgments for “know” responses in^41^). Possible explanations for these differences relate to factors such as the emotional content of the stimuli, the availability of prior knowledge during encoding, the retrieval testing procedure, and the degree of similarity of the sources associated with the different items^42–46^. Most relevant here, the strengths of the links between the different dimensions of the episode during encoding and the level of the participant’s active engagement are also thought to play a role^4,19,47,48^.

Here we assessed the relationship between recollection and source memory, following encoding in conditions that either entailed active behavior within rich and coherent spatio-temporal contexts (RW and VR), or merely involved sequences of unrelated trials, each comprising arbitrary pairs of objects and places-images (SL). Overall, we found that item- and source-memory were slightly better following encoding in SL compared to RW and VR (see Fig. 3a,c). At first, this may appear surprising given previous evidence that encoding in active and immersive conditions can strengthen memory^22,23^. However, it should be noticed that our participants engaged in many concurrent activities during RW and VR encoding, but not in SL, which may explain the overall lower memory performance (see also^4^). Unlike previous memory studies that made use of VR (e.g.^4,32,33^), in the current study the participants had to perform a series complex tasks throughout the encoding phase. The participants interacted with avatars, received instructions, stored and updated information about the items they had to buy and actively searched for the relevant shops in a large virtual town. The level of cognitive load and the complexity of the task were higher than in previous studies, when the participants typically focused attentional/cognitive resources on elements that were potentially relevant for the subsequent memory tests (e.g. exploring the VR, when later on asked to reproduce maps^49^ or to make judgments about crossing points^50^). The possible impact of concomitant activities was further exacerbated in the RW protocol. Further, when participants received the object-images, they may have focused on the phone screen and paid little attention to the surrounding environment. On the other hand, in RW, participants could perceive the environment for longer periods (several minutes before/after responding to the phone event), while in SL participants had only a few seconds to process the place-images. All these factors make it difficult to interpret the differences in the overall memory performance for objects and sources (see Fig. 3a,c).

This is why our main analyses focused on the relationship between source-memory and the subjective status of the memorized objects. The results showed that, in spite of the lowered overall performance, the contribution of incidentally encoded contextual information (places and times) was maintained across the three encoding contexts. The logistic-regression analyses showed that the likelihood of reporting the what-item as *Remember,* as opposed to *Familiar,* increased when the participants were able to discriminate with a high-level of confidence the associated place- or time-source (Fig. 3d). These results are in line with the notion that the availability of source information supports the subjective sense of recollection, as proposed by Tulving^15^ and postulated by dual-process models of episodic memory^39^. Nonetheless, it should also be noted that additional logistic analyses indicated that confident recognition of both time and place sources did not lead to any additional increase of *Remember* responses (see also^40^). Thus, while both spatial and temporal source signals contributed to subjective recollection, the underlying memory trace of the episode did not necessarily include an integrated representation of the what-where-when dimensions^31,48^: that is, in the framework of the current task, confident memory of one source-dimension was sufficient to explain the subjective sense of recollection.

Most importantly, we show that this relationship between subjective recollection and source-memory held across three vastly different encoding contexts. The investigation of episodic memory to real-world situations allows embracing two characterizing features of episodic memory: namely, the rich and multidimensional nature of the episodic events, and the strong association between the events and the person who experiences them^14,16^. Several previous studies addressed this by making use of wearable cameras (e.g.^35–37,51^). These protocols entail recording images of the everyday life of the participant and then make use of these scenes for subsequent memory assessments. Studies using wearable cameras revealed that the presentation of participant-specific scene images can strengthen episodic retrieval^35–37,51^ (see also^52,53^ for reviews, including the clinical relevance of these devices). However, other studies indicated that participants' memories of their own real-world experiences can be relatively poor^54^. These studies often made use of simple old/new judgment^54^ or more specific remember/know tasks^35^ but did not directly address the issue of the contribution of source memory to subjective recollection (but see^55,56^, for neuroimaging evidence of the role of the hippocampus in combining spatial and temporal information about everyday life events). Using a real-world procedure, Mazurek et al.^57^ directly tested the binding of what-where-when elements and the relationship between this and the subjective experience of retrieval (*remember* vs *know*). The results revealed no significant difference of correct what-where-when combinations between remembered vs. know episodes. These results contrast with the findings of the current RW protocol but it should be noted that the number of objects/events, the task at encoding and the spatio-temporal characteristics of the encoding context substantially differed between the two studies. Indeed, the main feature of our RW mobile-protocol was that it enabled us to generate events over long temporal windows (up to 3 weeks, vs*.* hours in^57^) and widespread spatial locations (kilometers, vs*.* meters in^57^).

The possibility of extending the duration of the encoding phase enabled us to address the hypothesis of scale invariance in long-term memory^58,59^. In the framework of the SIMPLE model^38^, we have previously shown that the relationship between temporal similarity and temporal-order reaction times holds across different encoding durations and retention intervals, in the seconds-to-hours range^5^. In the current study, we extended this approach to encoding periods lasting up to several weeks (RW protocol) and including retrieval cues based either on memorized what-items or contextual where-places. We found that the relationship between similarity and retrieval times holds across the three encoding contexts, consistent with the hypothesis that similar mechanisms contribute to order-retrieval over different scales (Fig. 4c). In the SIMPLE model, this would entail local interference arising when the two to-be-retrieved items are close within the “psychological space"^38^, see also below.

Our results also showed that the relationship between temporal similarity and retrieval times changed as a function of the cue employed to retrieve the temporal order of the events. The effect of temporal similarity was significantly larger when the participants judged the order of two objects than when they judged the order of two places. Notably, this was true also when we specifically tested places where the objects had been encoded (i.e. the *evTOloc* trials) and that, thus, had identical temporal similarity values as the corresponding object-trials. A straightforward explanation for this difference may be that the participants did not retain temporal information about places as good as they did for the objects. However, the effect of retrieval-cue was evident also for the VR protocol that—overall—lead to analogous order-retrieval performances, irrespective of cue dimension (see Fig. 4a, light-gray bars). The finding that the type of retrieval-cue modulates the relationship between temporal similarity and retrieval times suggests that factors other than chronological information contributed to the order judgment.

One proposal that considers the interplay between chronological and non-chronological signals during temporal retrieval comprises temporal context models^60^. These have been found to account for temporal-order performance following encoding in complex VR settings^33^. Temporal context models can also explain scale invariance, but they substantially differ from SIMPLE because they include context-specific temporal drifts and decays. Our current study was not designed to advocate between these models. However, the finding that SIMPLE accounted for the retrieval times across three contexts (RW, VR, SL) highlights the relevance of logarithmic scaling beside any context-specific effect. Moreover, our three encoding conditions substantially differed in terms of their spatio-temporal structure, i.e. a coherent continuum with different levels of complexity in RW and VR, vs. a set of unrelated trails in SL. These differences should impact processes relevant for context-based retrieval (e.g. event segmentation^10^), while our results showed analogous effects of temporal similarity in the three encoding protocols, see Fig. 4c.

Instead, we suggest that the impact of cue-type here may reflect the existence of a multi-dimensional space storing the memorized episodes. In the SIMPLE model, the “psychological space” determining the competition between neighboring events can comprise not only the chronological dimension, but also additional dimensions. In the initial formulation that considered the retrieval of simple stimuli, these additional dimensions would—for example—encode whether two word-items belonged to the same vs. different lists at encoding, or more complex hierarchical effects related to semantic content^38^. In the current study, a most important additional dimension concerned the place-related information that was associated with each event. Accordingly, events' similarity in the psychological space—and any resulting competition during retrieval—would entail not only chronological distances, but also interference caused by place information. In the framework of the SIMPLE model, Brown and colleagues^38^ proposed that the allocation of attentional resources during recall would bias the contribution of the different dimensions of the psychological space, “stretching out the relevant dimension, while simultaneously squashing it along another”. Here, this would correspond to the use of the different retrieval cues that in the *TOloc* condition (place-cues) would diminish any interference arising from the chronological distance and reduce the correlation between the temporal similarity values and the retrieval times (cf. Fig. 4c). Future studies may attempt to quantify the events' distance along the place-dimension, albeit this may be particularly difficult with naturalistic stimuli. Place-related information includes the real-world distance between events encoded in RW (e.g. see^55^), but also other factors such as the visual similarity between the place-images presented during retrieval^61,62^, as well as more complex parameters arising from the reconstructive mechanisms thought to govern the retrieval of complex, multi-dimensional naturalistic events^63,64^.

In conclusion, we investigated the contribution of contextual information to episodic memory, introducing a novel protocol based on mobile-phone technology that allowed us to study this relationship in a vast spatio-temporal, real-world context (see also^65^). The results highlighted the contribution of source memory to the subjective memory status of the tested-item and demonstrated the impact of temporal similarity on temporal-order retrieval irrespective of encoding context. These findings corroborate the link between the two characterizing aspects of episodic memory, namely the multi-dimensional nature of the memory trace and the subjective experience of retrieval, and they support proposals of temporal scale invariance in long-term memory. The study of cognitive functions outside the laboratory comes with many limitations that relate to the impossibility to control for a large number of factors likely to affect the processes under investigation. Nonetheless, naturalistic approaches are fundamental to bridge the gap between basic scientific knowledge and any application in the real world, including clinical practice, as well as putting to the test cognitive theories typically constructed on the basis of simple and stereotyped paradigms.

## Methods

### Participants

The experimental protocol involved three behavioral experiments (VR, RW, SL), each with a different group of 20 participants (age ranges, Male/Female ratios: VR: 23–42 years, 11/9; RW: 20–32 years, 8/12; SL: 21–33 years, 5/15). All participants were right-handed and had normal or corrected-to-normal vision. No neurological impairments or cognitive dysfunction was reported and all participants gave written informed consent. The study was approved by the Committee for Person Protection (CPP OUEST II, France, A02558-45), in accordance with the Declaration of Helsinki, and authorized by French “National Committee of Informatics and Freedom” (ref. N.: MMS/OTB/AR186736).

### Experimental procedures

Each of the three experiments comprised an encoding phase and a retrieval phase (see Figs. 1, 2). The retrieval tasks were analogous in the three experiments. The encoding phase comprised the memorization of 60 objects that, across the 3 experiments, were presented in vastly different spatio-temporal contexts, see Fig. 1 and Table 1. Pictures of the objects were taken from the “Massive Memory Object Categories” database^66^ and included common objects of different categories (e.g. tools, animals, food).

### Encoding protocols

In experiment 1 (Virtual Reality, VR), the pictures of the 60 objects were presented over a period of 45 min, while the participants actively explored a large-scale virtual town (see Fig. 1a). The virtual environment was viewed on a standard PC screen. The participants used the PC-keyboard to navigate within the town and to control various actions associated with a set of “missions”. The missions entailed collecting specific objects (e.g. T-shirts, medicines, shoes, fish, cigarettes, etc.) in specific virtual shops (e.g. clothes-shop, pharmacy, fishmonger, tobacconist, etc.). At unpredictable times, the participant was presented with the objects for the main memory task. The picture of the memory-object was shown in the lower part of the visual field and the participants used the keyboard to indicate whether they “liked/disliked” the object. The like/dislike judgment was included to make sure that the participant paid attention to the memory-objects. The retrieval phase took place the day after the encoding phase.

In experiment 2 (Real-World, RW), the same 60 objects were presented over a period of 3–17 days during the everyday life of the participants, via a dedicated mobile-phone application (Fig. 1b). The system acquires contextual data based on mobile-phone functionalities, including real-time GPS coordinates, current speed and motion direction and can make use of this real-time knowledge to make decisions about any information to be sent to the participant (see Suppl. Material, for the specific constraints used to decide when to send the object-images). During encoding, the only task of the participant was to look at the picture of the object sent on the mobile-phone and to respond whether they “liked/disliked” the object, via the mobile-phone interface. The mobile application signaled the object-events with a sound, plus a vibration notice. The retrieval phase took place at variable intervals after the encoding of the last object (hours to days, see Table 1).

In experiment 3 (Standard Laboratory, SL), the encoding phase followed a standard laboratory procedure, including the pairing of each object with the picture of a place on a computer screen in the laboratory. The encoding phase comprised 120 trials: 60 trials with place-images paired with the memory-objects and 60 trials comprising place-images only (see Fig. 1c). The place-images were obtained from the VR experiment and the task of the participant was again to indicate whether they “liked/disliked” the object. The SL encoding took place over a short period of 15 min and the retrieval started approx. 15 min after the end of the encoding phase. Further details about the three encoding procedures are provided in the Supplementary Materials.

### Retrieval tasks

The retrieval phase was identical in the three experiments and comprised two main tasks: explicit source retrieval and temporal-order judgment. The aim of the source retrieval task was to assess the participants’ memory for the 60 objects (what) and for the associated spatial (where) and temporal (when) context. Each trial included multiple phases, first assessing the subjective memory status of the object (*Remembered* object with where/when-context vs. *Familiar* object only) and then, explicitly testing memory for the where/when-sources using a two-alternative forced-choice procedure, plus confidence judgments (see Fig. 2a). The place-discrimination comprised the presentation of two place-images shown side-by-side. For the VR and SL experiments, one of the two images (the target) corresponded to the exact scene that was seen by the subject at the moment they encoded the object. For the RW experiment the images were obtained from Google-image and depicted the real-world location where the participant had received the object on their mobile-phone. The time-discrimination entailed the presentation of two time-windows (written text), one of which included the time when the memory-object was presented.

The second retrieval task comprised a temporal-order judgment. Each trial entailed the side-by-side presentation of two images (Fig. 2b). In different conditions, these retrieval cues included either what-objects or where-places. Specifically, the two images depicted either: two objects presented during encoding (*TOobj* task); two images of places, both associated with an object-presentation event (*ev-TOloc*); or (3) two images of places that were also visited/seen during the encoding phase, but that were not associated with any object-presentation event (*noe-TOloc*). The task of the participant was to report which of the two images (objects or places) referred to the time-point that had happened earlier during the encoding. The main data analysis fitted the retrieval reaction times with a temporal similarity model (SIMPLE^38,67^) that allowed us to assess scale invariance of the temporal-order retrieval taking into account both the temporal distance between each pairs of events during encoding and the retention times between encoding and retrieval (see Table 2). Further details about the retrieval tasks are provided in the Supplementary Materials.

**Table 2:** Temporal-order discrimination task.

	Virtual reality (VR)			Real world (RW)			Standard laboratory (SL)		
	*TOobj*	*ev-TOloc*	*noe-TOloc*	*TOobj*	*ev-TOloc*	*noe-TOloc*	*TOobj*	*ev-TOloc*	*noe-TOloc*
Temporal distance	0.7–42.4	0.7–42.4	0.5–42.7	6.4–2.5 × 10^4^	6.4–2.5 × 10^4^	0.7–2.3 × 10^4^	0.1–15.1	0.1–15.1	0.1–15.0
Temporal similarity	0.33–0.98	0.33–0.98	0.33–0.98	0.27–1.00	0.27–1.00	0.32–1.00	0.33–0.99	0.33–0.99	0.34–0.99
RT	2127 (260)	2559 (276)	2568 (300)	2092 (373)	2612 (371)	2843 (328)	1929 (384)	2461 (455)	2438 (492)
Accuracy	66.2 (2.7)	65.3 (1.5)	65.8 (1.8)	66.2 (2.4)	58.9 (2.5)	56.0 (3.5)	65.5 (2.7)	53.3 (2.0)	54.5 (1.8)

Temporal characteristics of the to-be-judged pairs of events during the temporal-order task and the average behavioral performance for the different conditions (TOobj, ev-TOloc, noe-TOloc) and encoding contexts (VR, RW, SL). *Temporal distance*: Time between the occurrence of the two events during encoding (range, in minutes); *Temporal similarity:* Similarity values computed according to the SIMPLE model (range, in similarity units [0–1], see “Methods” section). *RT:* Mean reaction times during retrieval (in ms, with standard error); *Accuracy:* Mean accuracy during retrieval (in %, with standard error).

### Data analysis

#### Explicit source retrieval

The aim of the explicit source-retrieval test was to assess the influence of source-memory on the subjective memory status of the object (*Rem* vs*. Fam*). The data analysis was carried out using mixed-effect binomial logistic regressions, implemented in Matlab R2017a (Mathworks, Inc.). Separately for the 3 experiments, the logistic models considered all single trials when an old/seen-object was correctly recognized either as *Rem* or *Fam*. The dependent variable was the *Rem* or *Fam* memory status of the object. The four predictors of interest comprised the place and time retrieval accuracies (irrespective of confidence) and the interaction between accuracy and confidence for the two sources. The factor “subject” was included as a random effect in the models.

An additional set of models tested for possible interactions between the two sources. Given the results of the main models, which showed that only high-confident correct source-responses predict *Rem*, the additional logistic model coded directly high-confident correct responses (= 1, vs. all other accuracy/confidence combinations = 0) for the two sources, plus the interaction between the two sources. The interaction term should highlight whether the ability to retrieve both place and time sources (correct and with high-confidence) leads to any further increase in the likelihood of responding *Rem* to the initial object-retrieval question.

#### Temporal-order judgments

The aim of the temporal-order task was to investigate scale-invariant mechanisms of temporal-order retrieval using the SIMPLE model proposed by Brown and colleagues^38^. For each single trial of each of the 3 experiments, we computed a “temporal similarity” value (TS) that takes into account the temporal distance between the two probes during encoding, as well as the time between the encoding of the probes and their retrieval. For instance, two events separated by a fixed temporal distance (e.g. 10 min) will be more “similar” (high TS) if they occurred a long time before retrieval, compared to two events with the same distance but occurring closer to the time of retrieval (lower TS). The TS were computed as:

$${\textbf{TS}}_{{\textbf{i,j}}} = \, \left( {{\textbf{T}}_{{\textbf{i}}} {\textbf{/T}}_{{\textbf{j}}} } \right)^{{\textbf{c}}} ,\,{\text{with}}\,{\text{T}}_{{\text{i}}} < {\text{ T}}_{{\text{j}}}$$

where T_i_ and T_j_ are the encoding-to-retrieval retention delays for the pair of events “i” and “j”. “c” is a power constant that here was computed as the inverse of the range of log-transformed T_i_ and T_j_^67^. The TSs take values between 0 and 1 irrespective of the range of temporal distances and retention delays (see Table 2, reporting the initial ranges of temporal distances and the corresponding TS ranges).

Following the computation of the TS for each trial, we used robust regressions implemented in Matlab 2017a to obtain the relationship between TSs and RTs for each participant separately for the three temporal-order conditions (*TOobj, ev-TOloc* and *noe-TOloc*), considering correct trials only. For statistical inference at the group level, the corresponding regression slopes (betas) were submitted to a 3 × 3 mixed-ANOVA with the factors: “cue-condition” (*TOobj, ev-TOloc*, *noe-TOloc*) and “experiment” (VR/RW/SL: between-groups). The ANOVA was carried out using SPSS (vers 21, IBM).

## Supplementary Information

Supplementary Informations.

## References

[1] McKenzie WA, Humphreys MS (1991). Recency effects in direct and indirect memory tasks. Mem. Cogn..

[2] Polyn SM, Erlikhman G, Kahana MJ (2011). Semantic cuing and the scale insensitivity of recency and contiguity. J. Exp. Psychol. Learn. Mem. Cogn..

[3] Gronau N, Neta M, Bar M (2008). Integrated contextual representation for objects’ identities and their locations. J. Cogn. Neurosci..

[4] Plancher G, Barra J, Orriols E, Piolino P (2013). The influence of action on episodic memory: A virtual reality study. Q. J. Exp. Psychol..

[5] Kwok SC, Macaluso E (2015). Scale invariance of temporal order discrimination using complex, naturalistic events. Cognition.

[6] Wu, C.-C., Wick, F. A. & Pomplun, M. Guidance of visual attention by semantic information in real-world scenes. *Front. Psychol.* **5**, 54 (2014). 10.3389/fpsyg.2014.00054 PMC3915098 24567724

[7] Henderson JM, Hayes TR (2018). Meaning guides attention in real-world scene images: Evidence from eye movements and meaning maps. J. Vis..

[8] Hannula DE, Libby LA, Yonelinas AP, Ranganath C (2013). Medial temporal lobe contributions to cued retrieval of items and contexts. Neuropsychologia.

[9] Hasson U, Furman O, Clark D, Dudai Y, Davachi L (2008). Enhanced intersubject correlations during movie viewing correlate with successful episodic encoding. Neuron.

[10] Kurby CA, Zacks JM (2008). Segmentation in the perception and memory of events. Trends Cogn. Sci..

[11] Burgess N, Maguire EA, O’Keefe J (2002). The human hippocampus and spatial and episodic memory. Neuron.

[12] Tulving, E. Episodic and Semantic Memory. In *Organization of Memory* (eds. Tulving, E. & Donaldson, W.) 381–403 (Academic Press, 1972).

[13] Chow TE, Westphal AJ, Rissman J (2018). Multi-voxel pattern classification differentiates personally experienced event memories from secondhand event knowledge. Neuroimage.

[14] Tulving E (1983). Elements of Episodic Memory.

[15] Tulving E (1985). Memory and consciousness. Can. Psychol./Psychol. Canad..

[16] Tulving E (2002). Episodic memory: From mind to brain. Annu. Rev. Psychol..

[17] Cunningham SJ, Turk DJ, Macdonald LM, Neil-Macrae C (2008). Yours or mine? Ownership and memory. Conscious. Cognit..

[18] Serbun SJ, Shih JY, Gutchess AH (2011). Memory for details with self-referencing. Memory.

[19] Engelkamp J, Perrig W (1986). Differential effects of imaginal and motor encoding on the recall of action phrases. Arch. Psychol. (Frankf).

[20] Helstrup T (1989). Loci for act recall: Contextual influence on the processing of action events. Psychol. Res.

[21] Holland SM, Smulders TV (2011). Do humans use episodic memory to solve a What–Where–When memory task?. Anim Cogn.

[22] Ruddle RA, Volkova E, Mohler B, Bülthoff HH (2011). The effect of landmark and body-based sensory information on route knowledge. Mem Cogn.

[23] Wallet G (2011). Virtual/real transfer of spatial knowledge: Benefit from visual fidelity provided in a virtual environment and impact of active navigation. Cyberpsychol. Behav. Soc. Netw..

[24] Kourtesis, P., Collina, S., Doumas, L. A. A. & MacPherson, S. E. Validation of the Virtual Reality Everyday Assessment Lab (VR-EAL): An immersive virtual reality neuropsychological battery with enhanced ecological validity. *J. Int. Neuropsychol. Soc. ***27**, 1–16 (2020). 10.1017/S1355617720000764 32772948

[25] Smith SA (2019). Virtual reality in episodic memory research: A review. Psychon Bull Rev.

[26] Yonelinas AP (2002). The nature of recollection and familiarity: A review of 30 years of research. J. Mem. Lang..

[27] Wixted JT (2007). Dual-process theory and signal-detection theory of recognition memory. Psychol. Rev..

[28] Howard MW, Eichenbaum H (2013). The hippocampus, time, and memory across scales. J. Exp. Psychol. Gen..

[29] Horner AJ, Bisby JA, Bush D, Lin W-J, Burgess N (2015). Evidence for holistic episodic recollection via hippocampal pattern completion. Nat. Commun..

[30] Ngo CT, Horner AJ, Newcombe NS, Olson IR (2019). Development of holistic episodic recollection. Psychol. Sci..

[31] Jebara, N., Orriols, E., Zaoui, M., Berthoz, A. & Piolino, P. Effects of enactment in episodic memory: A pilot virtual reality study with young and elderly adults. *Front. Aging Neurosci.* **6**, 338 (2014). 10.3389/fnagi.2014.00338 PMC4269133 25566069

[32] Persson BM, Ainge JA, O’Connor AR (2016). Disambiguating past events: Accurate source memory for time and context depends on different retrieval processes. Neurobiol. Learn. Mem..

[33] Horner AJ, Bisby JA, Wang A, Bogus K, Burgess N (2016). The role of spatial boundaries in shaping long-term event representations. Cognition.

[34] Baldassano C (2017). Discovering event structure in continuous narrative perception and memory. Neuron.

[35] Sellen, A. J.* et al.* Do Life-Logging Technologies Support Memory for the Past? An Experimental Study Using Sensecam. In *Proceedings of the SIGCHI Conference on Human Factors in Computing Systems,* 81–90 (Association for Computing Machinery, 2007). 10.1145/1240624.1240636.

[36] St. Jacques PL, Schacter DL (2013). Modifying memory: Selectively enhancing and updating personal memories for a museum tour by reactivating them. Psychol Sci.

[37] Selwood A, Bennett J, Conway MA, Loveday C, Kuchelmeister V (2020). Mnemoscape: Supporting older adults’ event memory using wearable camera photographs on an immersive interface. Gerontology.

[38] Brown GDA, Neath I, Chater N (2007). A temporal ratio model of memory. Psychol. Rev..

[39] Yonelinas AP (1999). The contribution of recollection and familiarity to recognition and source-memory judgments: A formal dual-process model and an analysis of receiver operating characteristics. J. Exp. Psychol. Learn. Mem. Cogn..

[40] Perfect TJ, Mayes AR, Downes JJ, Van Eijk R (1996). Does context discriminate recollection from familiarity in recognition memory?. Q. J. Exp. Psychol. Sect. A.

[41] Hicks JL, Marsh RL, Ritschel L (2002). The role of recollection and partial information in source monitoring. J. Exp. Psychol. Learn. Mem. Cogn..

[42] Donaldson W, Mackenzie TM, Underhill CF (1996). A comparison of recollective memory and source monitoring. Psychon. Bull. Rev..

[43] Hicks JL, Marsh RL (1999). Remember-know judgments can depend on how memory is tested. Psychon. Bull. Rev..

[44] Cook GI, Hicks JL, Marsh RL (2007). Source monitoring is not always enhanced for valenced material. Mem. Cognit..

[45] Rimmele U, Davachi L, Phelps EA (2012). Memory for time and place contributes to enhanced confidence in memories for emotional events. Emotion.

[46] DeWitt MR, Knight JB, Hicks JL, Ball BH (2012). The effects of prior knowledge on the encoding of episodic contextual details. Psychon Bull Rev.

[47] Graf P, Schacter DL (1989). Unitization and grouping mediate dissociations in memory for new associations. J. Exp. Psychol. Learn. Mem. Cogn..

[48] Diana RA, Yonelinas AP, Ranganath C (2008). The effects of unitization on familiarity-based source memory: Testing a behavioral prediction derived from neuroimaging data. J. Exp. Psychol. Learn. Mem. Cogn..

[49] Gaunet F, Vidal M, Kemeny A, Berthoz A (2001). Active, passive and snapshot exploration in a virtual environment: Influence on scene memory, reorientation and path memory. Cogn. Brain Res..

[50] Brunec IK, Ozubko JD, Barense MD, Moscovitch M (2017). Recollection-dependent memory for event duration in large-scale spatial navigation. Learn. Mem..

[51] Mair A, Poirier M, Conway MA (2017). Supporting older and younger adults’ memory for recent everyday events: A prospective sampling study using SenseCam. Conscious. Cogn..

[52] Dubourg L, Silva AR, Fitamen C, Moulin CJA, Souchay C (2016). SenseCam: A new tool for memory rehabilitation?. Rev. Neurol..

[53] Chow TE, Rissman J (2017). Neurocognitive mechanisms of real-world autobiographical memory retrieval: Insights from studies using wearable camera technology: Wearable cameras and autobiographical memory. Ann. N.Y Acad. Sci..

[54] Misra P, Marconi A, Peterson M, Kreiman G (2018). Minimal memory for details in real life events. Sci. Rep..

[55] Nielson, D. M., Smith, T. A., Sreekumar, V., Dennis, S. & Sederberg, P. B. Human hippocampus represents space and time during retrieval of real-world memories. *Proc. Natl. Acad. Sci. USA* **112**, 11078–11083 (2015). 10.1073/pnas.1507104112 PMC4568259 26283350

[56] Deuker L, Bellmund JL, Navarro Schröder T, Doeller CF (2016). An event map of memory space in the hippocampus. Elife.

[57] Mazurek, A., Bhoopathy, R. M., Read, J. C. A., Gallagher, P. & Smulders, T. V. Effects of age on a real-world What–Where–When memory task. *Front. Aging Neurosci.* **7**, 74 (2015). 10.3389/fnagi.2015.00074 PMC4435419 26042030

[58] Maylor EA, Chater N, Brown GDA (2001). Scale invariance in the retrieval of retrospective and prospective memories. Psychon. Bull. Rev..

[59] Moreton BJ, Ward G (2010). Time scale similarity and long-term memory for autobiographical events. Psychon. Bull. Rev..

[60] Howard MW, Kahana MJ (2002). A distributed representation of temporal context. J. Math. Psychol..

[61] Walker P, Hitch GJ, Duroe S (1993). The effect of visual similarity on short-term memory for spatial location: Implications for the capacity of visual short-term memory. Acta Physiol. (Oxf).

[62] Yago E, Ishai A (2006). Recognition memory is modulated by visual similarity. Neuroimage.

[63] Ranganath C, Ritchey M (2012). Two cortical systems for memory-guided behaviour. Nat. Rev. Neurosci..

[64] Frisoni M, Di Ghionno M, Guidotti R, Tosoni A, Sestieri C (2021). Reconstructive nature of temporal memory for movie scenes. Cognition.

[65] Piñeyro Salvidegoitia M (2019). Out and about: Subsequent memory effect captured in a natural outdoor environment with smartphone EEG. Psychophysiology.

[66] Konkle T, Brady TF, Alvarez GA, Oliva A (2010). Conceptual distinctiveness supports detailed visual long-term memory for real-world objects. J. Exp. Psychol. Gen..

[67] Neath, I. & Brown, G. D. A. SIMPLE: Further applications of a local distinctiveness model of memory. In *Psychology of Learning and Motivation,* vol. 46, 201–243 (Academic Press, 2006).

