# Supplementary material for: Memory for spatio-temporal contextual details during the retrieval of naturalistic episodes
Source: Sci Rep. 2021 Jul 16;11:14577. doi: 10.1038/s41598-021-93960-9 (PMC8285410; doi:10.1038/s41598-021-93960-9)
Supplement: Supplementary file 1 — Supplementary Informations. [file 41598_2021_93960_MOESM1_ESM.pdf]

SUPPLEMENTARY MATERIAL

Memory for spatio-temporal contextual details during the retrieval of naturalistic episodes

Samy Foudil, Claire Pleche, Emiliano Macaluso

I. Supplementary Methods

I.1. Encoding procedures

I.1.A.Virtual reality (VR)

I.1.B. Real-world (RW)

I.1.C. Standard Laboratory (SL)

I.2. Retrieval tasks

I.2.A. Explicit source retrieval

I.2.B. Temporal-order judgments

II. Supplementary Table

I. Supplementary Methods

I.1. Encoding procedures

I.1.A.Virtual reality (VR)

In the first experiment, the encoding of the 60 objects took place in a custom-build large-scale virtual town (Fig. 1a). The environment was built with the game engine Unity 3D (http://www.unity3D.com) and included several components and functionalities. The town extended over an area of 380 x 280 meters, comprising approx. 100 buildings. The participants used a PC-keyboard to navigate within the town and to control various actions associated with a set of "missions" they had to accomplish. The VR was viewed on a standard PC screen. The missions entailed collecting specific objects (e.g. T-shirt, medicines, shoes, fish, cigarettes, etc.) in specific virtual shops (e.g. clothes-shop, pharmacy, fishmonger, tobacconist, etc.). The participants received

the instructions about what object to collect either from avatars approaching them during the navigation or from "phone calls" comprising a ringing tone, followed by a voice giving the instructions. There were a total of 15 possible missions, each associated with one or more shops. The missions were triggered based on the position of the participant in the town, plus additional constraints (e.g. ensuring that there was at least 1.5 min between the beginning of two missions, unless the participant successfully completed the first mission within this time window). An additional list of 5 objects was given to the participant just before starting the navigation ("on-going" missions). Thus, the main task of the participant was to explore the town looking for the relevant shops where to collect the objects. Once inside the shop, the participant had to approach the counter, where an avatar greeted and prompted them to choose between 3 objects placed on the counter. These comprised the instructed-target (e.g. T-shirt), plus other objects pertinent with the shop (e.g. trousers and sweater, in the clothes-shop). The participant selected one of the objects and received 20 points if they chose the correct target-object, while 10 points were subtracted out if a wrong object was selected. The participant could enter in any shop and select any object at any time, but the 20 points were given only when collecting a currently relevant object. As soon as a target-object was collected, this became irrelevant. At any moment, the participant could check the number of points, the list of the 5 initial objects (on-going missions), as well as the time elapsed from the start of the navigation. The environment comprised many other features aiming to increase realism of the virtual experience, including a large number of moving avatars (ca. 250), moving cars, fountains with running water and environmental sounds.

While the participant explored the town aiming to complete the various missions, at unpredictable times, they were presented with the objects for the main memory task. Each of these events comprised a ringing tone, followed by the presentation a picture of the memory-object plus a choice display in the in the lower part of the visual field (cf. Fig. 1a). The navigation was blocked and the participant now used the left/right buttons of the keyboard to indicate whether they

"liked/disliked" the object. The presentation order of the objects was fix for all the participants, but the virtual location where these events occurred was specific for each participant depending on their exploration path. The timing of the events was also approximately the same for all subjects, but could slightly vary depending on the participants behavior. Before presenting the memory-object, there was a verification that the participant was not receiving any avatar/phone instruction and that they were not collecting any object in the shops. In these cases, the presentation of the memory-object was delayed of a few seconds, which lead to some variability in the timing of the events across participants.

The duration of the experience in the virtual town was 45 minutes and comprised the presentation of 60 memory-objects. By contrast, the number of triggered and completed missions varied across participants, again depending on individual behavior. Before starting the experiment, each participant underwent a 3-4 min training session in a smaller virtual environment. This aimed to familiarize the participants with the various aspects of the virtual experience (navigation, presentation the memory-objects, avatar/phone instructions, object collection, points system, etc.). The retrieval phase took place the day after the encoding phase, cf. also Tab. 1.

I.1.B.Real-world (RW)

In the second experiment, the encoding of the 60 objects took place in the real-world using a dedicated system, including an application installed on the mobile-phone of the participant (Fig. 1b). The system acquires contextual data based on mobile-phone functionalities, including real-time GPS coordinates, current speed and motion direction and can make use of this real-time knowledge to make decisions about any information to be sent to the participant. The system is based on 4 main components: 1) *The Mobile Application* that acquires User's information (GPS location and speed), shows the images of the memory-objects and records the participant's responses ("like/dislike" choice, see below); 2) *The SQL-Database* that stores User's activity logs, as well as the protocol features executed by Expert System; 3) *The Expert System* controls the experiment by

executing specific algorithms based on the experimental constraints and variables: here, "when and where" to send "what"-object, based on the participant's real-world position and previous history (see next paragraph for details); 4) The *InterCom System* that manages the data-communication between the mobile application, the SQL-Database and the Expert System. All together, this system makes it possible to acquire and produce reliable data taking into account various constraints (phone model, connection quality, satellite coverage, etc.) and to do this interactively in the daily life of the participants.

For the current study, the main constraint used by the *Expert System* to decide whether to send a new memory-object to the participant was their position in the real world. Specifically, on any given day, two objects could not be sent to the participant within a distance of less than 100 meters. Several other constraints were also implemented. These included: the speed of the participant that should not exceed 10 km/h, thus avoiding that the object was received while moving rapidly in a vehicle; the time between two successive events, which should not be shorter than 5 minutes; the reliability of the GPS data (spatial resolution < 5 m); and that Google-images of the current location should be available (see also below). The system was active between 8.00 and 20.00 and no data were acquired (or objects sent) during the night. Because of these different constraints, the time needed to trigger the 60 memory-objects events varied substantially between participants (3-17 days, see Tab. 1).

On the day of the inclusion, the mobile application was installed on the participant's personal mobile-phone. They were told that they would receive pictures of objects during the following days and the study could last several weeks depending on their movements in the real world. They were also told that they would have to come back to the laboratory to do some test concerning the objects, but were not informed about what type of tests (cf. retrieval tasks, below). During the encoding phase, the only task of the participant was to look at the picture of the object sent on the mobile-phone and to respond whether they "liked/disliked" the object, via the mobile-phone

interface. The mobile application signaled the object-events with a sound, plus a vibration notice. If the system did not record any response within 2 min following the object-delivery, the event was repeated at a later time point. The time and the location when the participant responded were recorded and used for the memory retrieval tasks, as well as new constraints for the triggering subsequent encoding events (cf. above). The retrieval phase took place at variable intervals after the encoding of the last object (hours to days, see Tab. 1).

I.1.C.Standard Laboratory (SL)

The encoding phase of the third experiment comprised a standard object-place pairing on a computer screen in the laboratory. The encoding phase comprised 120 trials: 60 trials with place-images paired with the memory-objects and 60 trials comprising place-images only (see Fig. 1c). The place-images were obtained from the VR experiment, thus depicting first-person snapshots of the virtual town. The images were shown in the center of the screen for 6 seconds. When the trial also included a memory-object, this appeared in the lower part of the place-image, 3 sec after the trial onset. Together with the picture of the object, a written instruction prompted the participant to report whether they "liked/disliked" the object. Object and instructions were displayed for 3 seconds and the participant responded using the left/right arrows of the PC keyboard. Trials with/without memory-objects were randomly intermixed. The inter-trial interval was 1500 ms. The total duration of the encoding phase was 15 min. The retrieval phase started approx. 15 minutes after the end of the encoding phase (see Tab. 1). During the retention period the participants watched a short movie on the PC screen.

I.2. Retrieval tasks

The organization of the memory retrieval tasks was the same in the 3 experiments. This entailed a first block when the participants were tested for object- and source- (place/time) memory,

followed by two blocks when they performed temporal-order judgment tasks (objects, and places with/without object-events).

I.2.A. Explicit source retrieval

The explicitly source retrieval task comprised multiple phases (see Fig. 2a). Each trial started with the presentation of the picture of an object. In 60 trials the object-picture was identical to the one seen during encoding, while on the other 60 trials the picture included an object of the same category as one of the memorized-objects, but depicting a different exemplar (memory foils). The task of the participant was to choose one of 3 possible responses: a) I have seen this object and I have some memory of the place/time when this happened; b) The object is familiar, but cannot remember when/where I saw it; c) I have not seen the object. We label these three response-types as "Remembered" (*Rem*), "Familiar" (*Fam*) and "New" (*New*). If the object was seen during encoding and the participant responded *Rem* or *Fam*, the trial went on testing for when/where source-memory and confidence. If the object was not seen during encoding or the participant responded "New" to a seen object, the trial was terminated. The image of the object remained on the screen for 3 seconds and was followed by a 2 to 3 seconds inter-trial interval. The participant responded using the left/up/right arrows-keys of the PC keyboard.

The source-memory test comprised four phases: place-test and place-confidence, time-test and time-confidence. In half of the trials the place-test (followed by place-confidence) was tested first, and in the other half of the trials time (and time-confidence) was tested first. The place-test comprised the presentation of two place-images shown side-by-side (see Fig. 2a). For the VR and SL experiments, one of the two images (the target) corresponded to the exact scene that was seen by the subject at the moment they encoded the object. The second image also corresponded to a scene that had been seen during the encoding phase: for the VR experiment this was a snapshot taken during the navigation, but not associated with any memory-object event; for SL, it was one of the 60 place-images presented without any objects during the encoding phase (cf. above, and Fig. 1c).

For the RW experiment the images were obtained from Google-image and depicted the real-world location where the participant had received the object on their mobile-phone (see Fig. 2a). We made use of the phone orientation data to obtain event-specific view angles, but always checked that this corresponded to a recognizable place (e.g. not just showing a plain wall and, in this case, we manually modified the view angle). Nonetheless, it should be noticed that while in the VR and SL protocols the place-images presented at retrieval were identical to those seen at encoding, this was not the case in the RW protocol. The images available on Google-image depict the location at one specific moment that does not correspond to when the participant was there. Thus, the images presented at retrieval included different objects/people compared to what the participant experienced during encoding, possibly also displaying a different time of the day, season and weather conditions. As for the VR and SL protocol, the foil image also showed a place that was visited by the participant (identified using the individual GPS data), but where no object-event took place.

In all three experiments, the images of the two places were displayed for 3 seconds, together with the image of the currently relevant object and the text instruction: "What place ?" (see Fig. 2a). The participant reported at which of the two places they had received the object by pressing the left/right arrow-keys of the keyboard. After a short interval (blank screen, 2-3 sec), the question "Are you sure (place) ?" appeared on the screen and the participant had to choose between the two responses: "little" vs. "a lot", using the left/right arrow-keys of the keyboard. The confidence-judgment screen was presented for 2 sec, followed by a 2-3 sec inter-trial interval.

The time-test followed a similar procedure, comprising first a 2-alternative forced-choice between two possible time-windows, followed by the time-confidence judgment; see Figure 2a. The durations of the encoding phases were very different in the 3 experiments (cf. Tab. 1), implying different time-windows across the experiments for the time-test at retrieval. For the VR experiment, we defined 10 windows of 5 min each (e.g. "25 - 30 min"); for the RW experiment, the time-

windows corresponded to the days of the encoding phase (e.g. "day 3"); for the SL experiment, we defined 5 windows of 3 min each (e.g. "6 - 9 min"). The time-test started with a display showing the two time-windows: one including the time of the presentation of the memory-object during the encoding phase and the second corresponding to the time when the place-foil image was taken (cf. above). Thus, during the source-tests the "where" and "when" information was always congruent/correct both for the target and for the foil.

The text with the two time-windows was displayed for 3 seconds, together with the image of the currently relevant object and the text instruction: "What time ?". The participant pressed the left/right arrow-keys of the keyboard to indicate the window corresponding to the encoding time of the memory-object. After a short interval (blank screen, 2-3 sec), the question "Are you sure (time) ?" appeared on the screen and the participant had to choose between the two responses: "little" vs. "a lot", using the left/right arrow-keys. The confidence-judgment screen was presented for 2 sec, followed by a 2-3 sec inter-trial interval.

Accordingly, for each seen-object that was recognized as "Remembered" or "Familiar" we obtained 5 responses indicating: 1) the subjective memory status (*Rem*/*Fam*); 2) discrimination of the place-source (correct/wrong); 3) place-confidence (high/low); 4) discrimination of the time-source (correct/wrong); 5) time-confidence (high/low). These allowed us to assess how the availability (and confidence) of source-information contributed to the subjective memory status of the object.

<u>I.2.B. Temporal-order judgments</u>

Following the first retrieval block (explicit source-retrieval, cf. above), the participants performed two additional memory testing blocks, both comprising temporal-order judgments tasks (Fig. 2b). The procedure was the same for the three experiments. The first temporal-order block comprised 30 trials. On each trial, the participant was presented with the pictures of two objects that they had seen during encoding (temporal-order task, with object-cues: *TOobj* task). Each of the 60

encoded-objects was used only once. The pictures were shown side-by-side and the participant had to report which of the two objects they had seen before during the encoding phase. The task instruction ("Seen first ?") was displayed above the two objects and the responses were made with the left/right arrow-keys of the keyboard. The display was presented for 4 seconds, followed by a variable inter-trial interval (2-3 sec). The temporal distance between the encoding time-points of the two objects presented at retrieval varied parametrically between trials (see also "data analysis" section).

The second temporal-order block included 60 trials. All trials comprised the presentation of two place-images displayed side-by-side (*TOloc* task, see Fig. 2b). On half of the trials, both images depicted places associated with a memory-object event (place-cue, with object-event: *ev-TOloc*), while in the other 30 trials they depicted places that were also visited/seen during encoding but that were not associated with any object presentation event (place-cue, without object-event: *noe-TOloc*). Again, across trials, the temporal distance between the events at encoding varied parametrically. For the 30 *ev-TOloc* trials, the events' pairs were the same as the ones used for the *TOobj* task, thus the temporal distances tested in the *TOobj* and *ev-TOloc* conditions were identical (see also Tab. 2). For the *noe-TOloc* trials, the selection of the pairs was done with the aim of obtaining a range of temporal distances comparable with those used for the *ev-TOloc* trials. For this, for each subject, the pairing between the *noe-TOloc* place-images was repeated until the corresponding set of temporal distances was not significantly different from that obtained for the *ev-TOloc* trials ($p > 0.5$). The presentation order of the *ev-TOloc* and *noe-TOloc* trials was intermixed and randomized. The images of the places were displayed for 4 seconds, together with the task instruction ("Seen first ?"). The participants responded with left/right button-presses. The inter-trial interval was 2-3 sec.

II. Supplementary Table

	Virtual Reality (VR)		Real World (RW)		Standard Laboratory (SL)	
OBJECTS						
**Accuracy *Old***	80.5 (2.0)		73.3 (3.1)		87.0 (2.4)	
**Accuracy *New***	74.7 (3.0)		76.7 (3.7)		91.0 (1.5)	
**Ratio *Rem/Fam***	0.66 (0.05)		0.54 (0.06)		0.62 (0.09)	
SOURCES	*High conf.*	*Low conf.*	*High conf.*	*Low conf.*	*High conf.*	*Low conf.*
**Accuracy *Place***	57.8 (3.9) *	*50.7 (1.9)*	71.6(4.7) *	*50.7 (2.7)*	76.5 (3.2) *	*48.0 (2.1)*
**Accuracy *Time***	61.2 (2.6) *	*49.7 (2.5)*	69.7 (4.0) *	*48.7 (2.8)*	67.7 (2.5) *	56.7 (2.9) *

Table S1. Accuracy of the object- and source-retrieval for the explicit source retrieval task. *Objects*: Mean accuracy (in %, with standard errors) for Old/seen objects irrespective of "Remember" or "Familiar" responses, for New/unseen objects (memory foils) and the ratio between Remember/Familiar responses for correct old/seen trials. *Sources*: Mean accuracy of the source discrimination (in %, with standard errors), as a function of the subsequent confident judgment (high/low confidence), see also Fig. 3. *: accuracy of source-discrimination significantly above the 50% chance level.
